# Structure and composition of prey remains of Cinereous Vultures (*Aegypius monachus*) from an adaptation aviary in Bulgaria

**DOI:** 10.3897/BDJ.14.e184922

**Published:** 2026-04-14

**Authors:** Rusko Petrov, Monika Razhdanova, Angelina Yaneva, Anton Stamenov

**Affiliations:** 1 Trakia University, Stara Zagora, Bulgaria Trakia University Stara Zagora Bulgaria; 2 Green Balkans - Stara Zagora NGO, Stara Zagora, Bulgaria Green Balkans - Stara Zagora NGO Stara Zagora Bulgaria; 3 Bulgarian Society for the Protection of Birds, Sofia, Bulgaria Bulgarian Society for the Protection of Birds Sofia Bulgaria

**Keywords:** *Aegypius monachus*, prey remains, structure and composition, domestic animals

## Abstract

This article presents the results of a study on the shape and composition of Cinereous vulture (*Aegypius monachus*) pellets. A total of 501 pellets were collected during two different seasons – 125 on 24 September 2025 and 376 on 10 March 2025. The birds from which the materials were collected were raised in a closed adaptation aviary near the town of Madzharovo and were fed with the carcasses of various domestic animals - Karakachan sheep (*Ovis aries*), horses (*Equus ferus caballus*), Native Bulgarian goat (*Capra hircus*), Shorthorn Rhodopian Cattle (*Bos taurus*), Bulgarian Gray Cattle, Bulgarian Black and White Cattle, European Bison (*Bison bonasus*) and domestic pigs (*Sus domesticus*). The study reveals the shape and composition of each of the pellets during the two collection periods and the results aim to highlight the similarity between them and the existence of a common type of pellet for the Cinereous vulture. This study also aims to determine what part of the food composition remains in the pellet after the carcass has been processed. After analysis, two types of residue were found in them - nutritional and non-nutritional. The nutritional residues consisted of fur and skin - as the highest percentage, stomach contents, bones, hooves, horns and feathers from vultures, while the non-nutritional residues consisted mainly of stones and sticks - as the most significant percentage, leaves, construction foam and plastic (sisal and cord).

## Introduction

The Cinerious vulture (*Aegypius monachus*) is the largest Eurasian raptor species with a population spanning from Portugal to Russia's Far East which numbers 8,400-11,400 pairs globally (Reading et al. 2020, BirdLife International 2021). The only remnant species population on the Balkans is found in Dadia-Soufli-Lefkimi Forest National Park (hereafter Dadia NP) and has been estimated at 21–35 pairs (Botha et al. 2017). In Bulgaria, the Cinereous vulture population marked a large decline in the 20^th^ century to fully disappear from the country in 1993 when the last confirmed breeding was recorded (Marin et al. 1998). Its extinction was a result of a number of factors, such as the persecution of birds of prey as "harmful game" in the mid-20^th^ century, the use of poisonous baits to control predators, habitat destruction, a significant reduction in the food base and а campaign to poison predators in the period 1958–1970, which continued by inertia until 1990s. This had the greatest impact on the population of the Cinereous vulture, as well as other birds of prey, such as the Bearded vulture, the Egyptian vulture, the Griffon vulture and the Imperial eagle (Lazarova et al. 2020, Dobrev et al. 2021). The species re-introduction on the Balkans was initiated in 2015 with the start of the project "A Bright Future for the Black Vulture" LIFE14 NAT/BG/649 and continued in the framework of the "Bearded Vulture LIFE" project (project No. 101113869 LIFE22-NAT-BG-Bearded Vulture LIFE) (Ivanov et al. 2023). The first release of Cinereous vultures in Bulgaria took place in the Kotlenska Planina protected area near Kotel in 2018 and continued with releases in the Sinite Kamani Nature Park in 2019-2022 and in the Vrachanski Balkan Nature Park in 2020-2022. A total of 72 individuals have been released at two sites in order to trigger species comeback. As a result, local populations have been successfully established in two different regions in Bulgaria – the Eastern Balkan and the Vrachanski Balkan and the Cinereous vultures in both release sites attract birds from neighbouring colonies in Greece and Turkey (Ivanov et al. 2023). In the meantime, a separate translocation project had started in the Eastern Rhodopes, Bulgaria enforcing species conservation actions in the country and adding up more than 40 individuals released in the wild so far (Dobrev et al. 2020, Dobromir Dobrev, pers. comm.).

The Cinereous vulture feeds mainly on the carcasses of domestic and wild mammals, preferring hard body tissues, such as bones, cartilage and tendons (Costillo et al. 2007a, Costillo et al. 2007b, Yamaç and Günyel 2010, Rajabova and Karimov 2021). It searches for food in groups or individually and is considered a resident species — the movement of individual birds is considered wandering (Coleman and Fraser 1987, Andevski and Tavares 2017, Wahl et al. 2024). The species' nesting habitat requirements are open areas with extensive livestock farming or places where slaughterhouse waste or animal carcasses are disposed of (Kelly et al. 2007, Yamaç and Bilgin 2012, Hill et al. 2022). Individuals reach sexual maturity at 4-5 years of age. Females lay one egg between January and March, with the young hatching in March-April and remaining in the nest for more than 3 months. After leaving the nest, they continue to live in a family group with their parents until they reach sexual maturity (Ivanov et al. 2023).

In Bulgaria, the Cinereous vulture inhabits a wide range of habitats where it feeds mainly on livestock (Batbayar et al. 2006), but it can also consist of diverse food sources such as sheep, carnivores and even tortoises (Skartsi et al. 2015, Karimov and Guliyev 2017). In Turkey, for example, they feed on wild boars (*Sus scrofa*), predators such as wolves (*Canis lupus*) and Red foxes (*Vulpes vulpes*) and even plants (Yamaç and Günyel 2010). Each raptor species has developed its own hunting strategy which defines its dietary diversity. Pellets regurgitation is the process of expelling substances which can not be digested (mainly bones, feathers, fur, chitinous structures, seeds, artificial materials) and this process has an important effect over birds’ health and nutrition (Sutherland et al. 2004, Barbosa et al. 2021). The morphological structure of pellets from captive Cinereous vultures has never been studied before; therefore, the aim of the current study is to present the amount of pellets produced by vultures kept in captivity, to reveal their size and composition and to record any specifics which might be used when collecting samples in the wild.

## Materials and Methods

**Figure 1.**
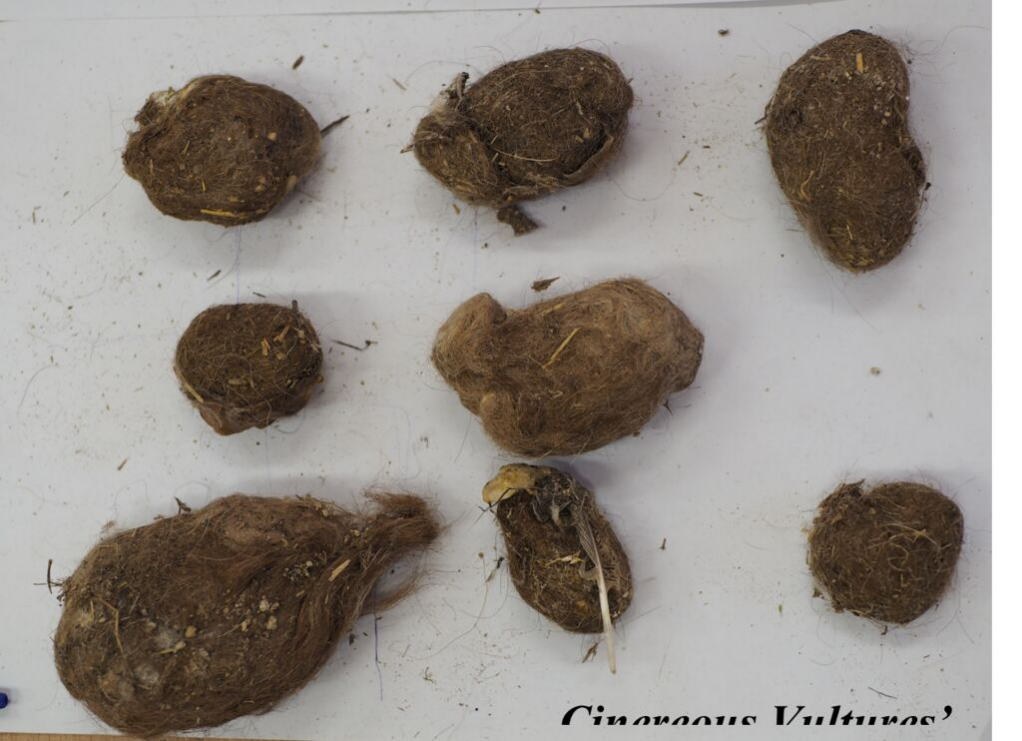
Cinereous Vultures' pellets.

**Figure 2.**
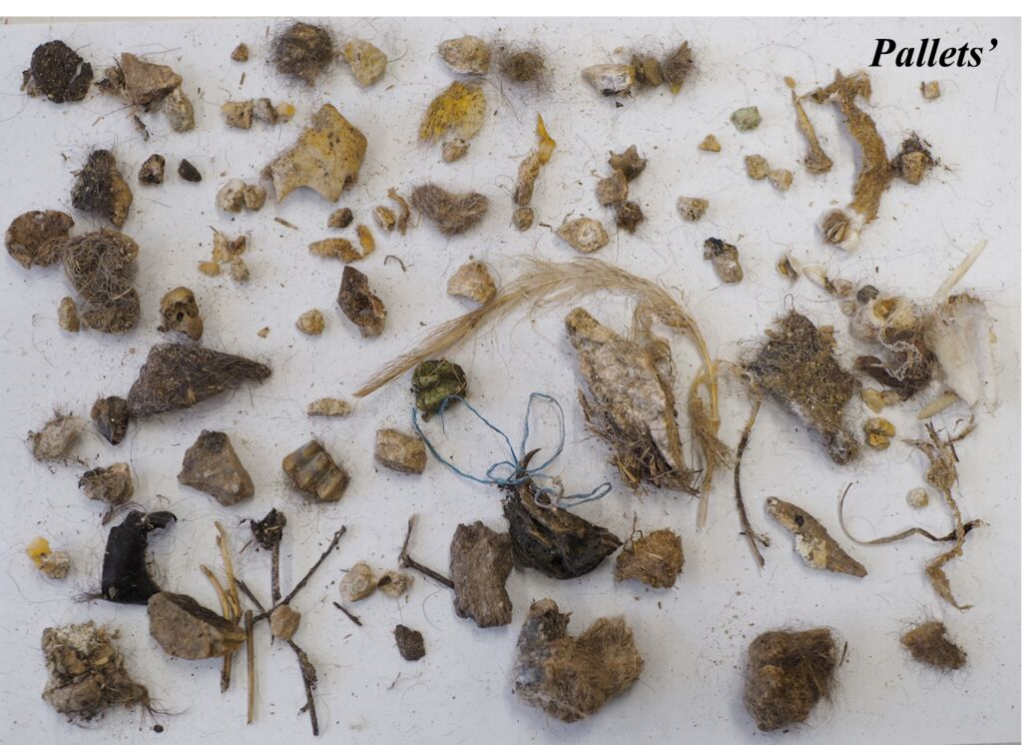
Cinereous Vulture pellet composition, January-March 2025.

**Figure 3.**
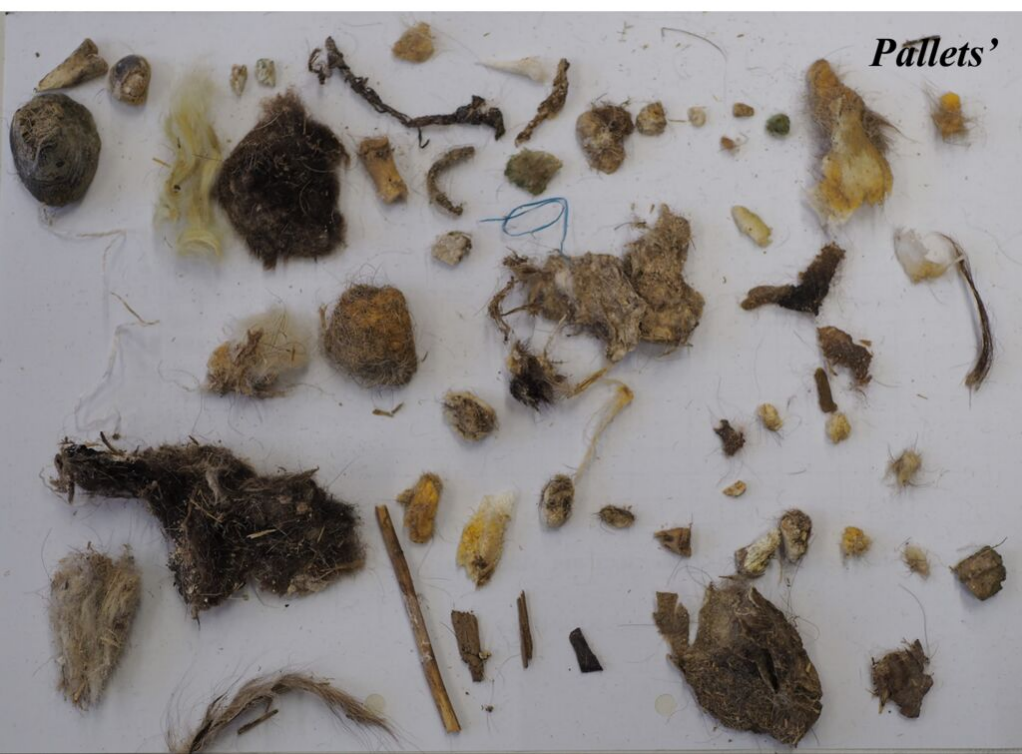
Cinereous vulture pellet composition, April-September 2025.

The study was carried out throughout 2025 in the Cinereous vulture adaptation aviary near Madzharovo, Eastern Rhodopes, Bulgaria. In this period, all the pellets remaining after vulture feedings inside the adaptation aviary were collected in two different periods – 121 from the food provision period from April to September 2025 and 376 from the period from January to March 2025 (Figs 1, 2, 3).

All collected pellets (n = 501) were stored, checked and measured separately. Several types of pellet structures were identified: square, round, oval, flat, triangular, rectangular and spherical.

Food items inside of each pellet were identified down to a species taxonomic level. All food items provided to the vultures in the adaptation aviary were of known origin and species - they were fed exclusively with carcasses of domestic animals and the examination of the pellets revealed parts of various domestic animals, including Karakachan sheep (*Ovis aries*), horses (*Equus ferus caballus*), Native Bulgarian goat (*Capra hircus*), Shorthorn Rhodopian Cattle (*Bos taurus*), Bulgarian Gray Cattle, Bulgarian Black and White Cattle, European Bison (*Bison bonasus*) and domestic pigs (*Sus domesticus*). As vultures were provided also with soft tissues and meat which were not be found in the pellets, we have not accounted for this in our research.

Separately, non-nutritional components found inside of the pellets such as stones, sticks, leaves, construction foam and plastic (sisal and cord) were also recorded.

The examined material was divided in respect to the season in which it was collected, the first batch being from the winter period from January to March and the second from the spring/summer period, from April to September.

## Results

**Figure 4.**
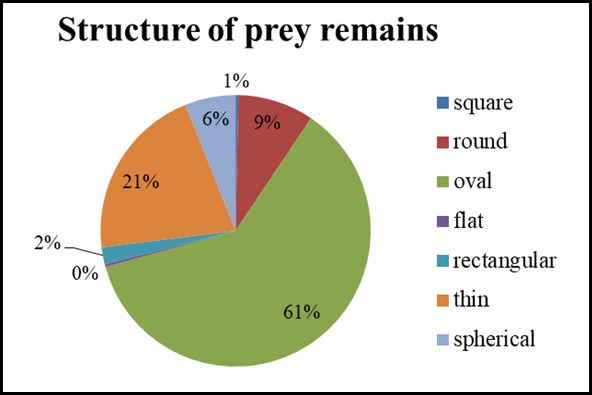
Structure of prey remains, January-March 2025.

**Figure 5.**
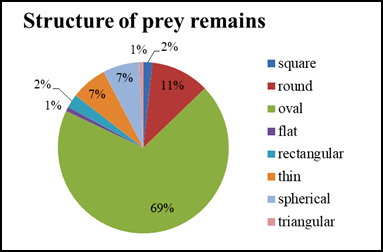
Structure of prey remains, April-September 2025.

In spring/summer, the prey remains were lighter in colour, while in the winter, they were darker. The carcasses vary in size, but in both seasons, one shape is most common — oval, accounting for an average of 65% of the total number (Figs 4, 5).

**Figure 6.**
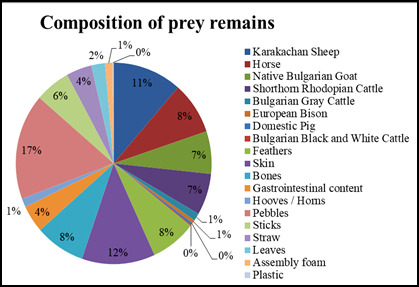
Composition of prey remains, January-March 2025.

**Figure 7.**
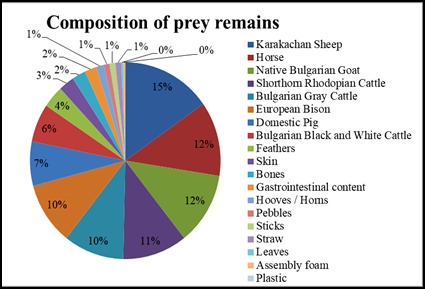
Composition of prey remains, April-September 2025.

The largest percentage of the pellets consists of parts of Karakachan sheep and horses, with a higher percentage of Karakachan sheep remains found in spring/summer (15%) and a higher percentage of horse remains found in winter (17%). The least remains found are from European bison, Bulgarian Black and White cattle and domestic pigs. In almost every pellet, breast and belly feathers of Cinereous vultures were found. They had fallen out of the vultures themselves and landed on the ground and on the carcasses they had eaten (Figs 6, 7).

The longest feather in the prey remains collected in the spring/summer was 9 cm and in those collected in the winter, it was 14.5 cm. A large number of undigested bones were found, the largest of which were two - 5.6 cm from the spring/summer and 4 cm from the winter. Undigested particles of hooves and horns of small and large ruminants were found, with the longest hoof particle found in the spring/summer pellets measuring 3.3 cm. Small pieces of skin were also found amongst the animal carcass remains, the longest of which reached 6.3 cm. A kestrel (*Falco tinnunculus*) leg was also found in the April-September pellets.

The non-nutritional composition of the prey remains consists mainly of stones, straw and sticks, with a higher straw content in the spring/summer pellets. This is due to the different diet of the animals in winter and spring/summer, which includes straw.

The largest stone, measuring 6 cm, was found in the pellets collected in spring/summer. The two longest sticks found in each season are 6.5 cm from the spring/summer and 5.8 cm from the winter, which is also 1.5 cm wide. Traces of construction foam and plastic - sisal and cord - were also found in the pellets, with the longest piece being 25 cm. All non-nutritional elements in the pellets were removed from the core of the pellets and anything stuck to the sides was not taken into account.

## Discussion

The study reveals key features in the morphology and composition of the prey remains of Cinereous vultures (*Aegypius monachus*) reared in captivity, which reflect seasonal adaptations to the feeding regime and environment in an adaptation aviary near Madzharovo. Seasonal differences in colour — lighter in spring/summer and darker in winter — are probably due to varying degrees of oxidation of nutritional residues and food moisture. The predominance of the oval shape (65% of cases on average) is due to muscle contractions that standardise the shape regardless of size variations.

The composition of the pellets highlights the role of the species as an effective scavenger, removing unassimilated parts of domestic animal carcasses – mainly hair, bones, stomach contents and skin. The beak of the Cinereous vulture is made for ripping skin and tendons. Hence, they feed mainly on muscles and small peripheral scraps of meat and skin (Moreno-Opo et al. 2010b) making ungulates’ skin an important nutrition unlike Griffon vultures (*Gyps fulvus*) (Margalida et al. 2011) or Egyptian vultures (*Neophron percnopterus*) (Dobrev et al. 2015). The predominance of remains of Karakachan sheep (15% in spring/summer) and horses (17% in winter) reflects their breeding in the area. In Spain, for example, pellets collected close to active nests and perches showed that they contain mainly pigs (54.6%), sheep/goats (37.1%) and cattle (8.3%) (Moreno-Opo et al. 2010a). However, some remains, for example, the domestic rabbit (*Oryсtolagus ciniculus domesticus*) were not found in the pellets although such items were provided to the birds (Dobromir Dobrev, pers. comm.). This is an important finding and might imply that even some hard items might be completely digested by the birds depending obviously on the prey item consumed. The feathers (up to 14.5 cm in winter) and bones (up to 5.6 cm in spring/summer) found, including a kestrel (*Falco tinnunculus*) leg, indicate opportunistic feeding, in which vultures ingest their own feathers from the ground and smaller raptors. Another explanation is that, even in captivity, vultures can access food remains scavenged and/or preyed at the close vicinity of the aviary. These findings illustrate the physiological limits of triptysis in Accipitridae, where hard structures such as hooves (up to 3.3 cm) and horns remain undigested. Some of the stones were likely swallowed accidentally or to aid digestion.

Non-nutritional particles – stones (up to 6 cm), sticks (up to 6.5 cm), straw and anthropogenic materials (plastic up to 25 cm, construction foam) – pose a significant risk to the Cinerious vulture. This finding has a significance to maintenance and construction of bird aviaries where all aging and disintegrating artificial materials must be secured away from birds' access. The high proportion of stones suggests geophagy to aid digestion, while the predominance of straw in spring/summer is explained by the domestic animals' winter feeding regime. However, the presence of plastic (sisal, cord and foam) indicates serious contamination of feeding areas, which can lead to blockage of the gastrointestinal tract or toxic accumulation. The results highlight the effectiveness of closed adaptation enclosures for study and preparation for re-introduction, with feeding on domestic carcasses minimising the risk of zoonoses. However, seasonal variations require protocol optimisation — more variety in winter to balance energy intake. Future studies should include chemical analysis of contaminants, microbiological screening and comparison with wild populations to assess effects on reproductive success and health.

## Conclusions

The prey remains of Cinereous vultures (*Aegypius monachus*) in an adaptation aviary near Madzharovo show seasonal differences – lighter with straw in spring/summer and darker with more horse remains in winter, with the oval shape dominating in 65% of cases. The main composition includes undigested parts of domestic animals (goat 15% in spring/summer, horse 17% in winter), own feathers up to 14.5 cm, bones up to 5.6 cm, hooves up to 3.3 cm and opportunistic finds such as a kestrel's leg. Non-nutritional finds— stones up to 6 cm, sticks up to 6.5 cm and plastic up to 25 cm — point to geophagy and anthropogenic pollution, which poses a health risk. Closed enclosures, such as adaptation aviaries, are an effective model for re-introduction, but require optimisation of feeding and waste control. The results highlight the sanitary role of the species and the need for measures to protect it.
